# Draft genome sequences of four strains of *Ralstonia pseudosolanacearum*

**DOI:** 10.1128/mra.00803-26

**Published:** 2026-08-10

**Authors:** Shunsuke Takahashi, Shogo Konishi, Jun Ogata, Takayoshi Hisada

**Affiliations:** 1 TechnoSuruga Laboratory Co., Ltd., Shizuoka, Japan; University of Strathclyde, Glasgow, United Kingdom

**Keywords:** *Ralstonia pseudosolanacearum*, draft genome sequence

## Abstract

We report the draft genome sequences of four *Ralstonia pseudosolanacearum* strains obtained from the NARO Genebank, Japan. Illumina MiSeq i100 sequencing generated 2 × 300 bp paired-end reads. The draft assemblies ranged from 5.67 to 5.86 Mb, with GC contents of 66.9%–67.0%.

## ANNOUNCEMENT

*Ralstonia pseudosolanacearum* is a member of the *Ralstonia solanacearum* species complex and is a major causal agent of bacterial wilt affecting a wide range of economically important crops worldwide (1, 2). Genomic resources from multiple strains support comparative studies of intraspecific diversity within this species (3–5).

In this study, four strains (MAFF106604, MAFF211270, MAFF730132, and MAFF730133) were obtained from the NARO Genebank (National Agriculture and Food Research Organization, Japan) as lyophilized cultures in ampoules under the taxonomic designation *Ralstonia pseudosolanacearum*. According to the NARO Genebank records, these strains were originally isolated from tomato at various locations in Japan (Table 1). The lyophilized cultures were rehydrated in 100 μL of sterile distilled water, and the resulting suspensions were streaked onto casamino acid-peptone-glucose (CPG) agar (6). After aerobic incubation at 28°C for 1 day, the cultures were used for genomic DNA extraction. Genomic DNA was extracted using the ISOSPIN Fecal DNA Kit (Nippon Gene, Tokyo, Japan) according to the manufacturer’s instructions. Sequencing libraries were prepared using the Nextera DNA Flex Library Preparation Kit (Illumina, USA) according to the manufacturer’s instructions. Paired-end sequencing (2 × 300 bp) was performed on an Illumina MiSeq i100 platform using the MiSeq i100 Series 25M Reagent Kit (600 cycles; Illumina).

**TABLE 1:** Genome statistics of *Ralstonia pseudosolanacearum* strains reported in this study

Strain	Raw read pairs^*a*^	Coverage (×)	Genome size (bp)	Contigs	N50 (bp)	GC content (%)	CDS	tRNA	Accession	Isolation location and year
MAFF106604	3,945,522	138.0	5,672,609	108	120,246	67.0	4,932	60	BAAKSV010000000	Kumamoto Prefecture, Japan (1993)
MAFF211270	3,897,044	138.4	5,693,451	104	126,134	67.0	4,951	60	BAAKSW010000000	Shizuoka Prefecture, Japan (1995)
MAFF730132	4,406,426	136.2	5,806,443	227	86,381	66.9	5,089	60	BAAKSX010000000	Shizuoka Prefecture, Japan (1982)
MAFF730133	3,776,459	134.8	5,855,681	350	95,983	66.9	5,084	61	BAAKSY010000000	Hyogo Prefecture, Japan (1982)

^*a*^ The numbers of read pairs represent the raw sequencing output before quality filtering and subsampling. Genome assemblies were generated using 1.5 million randomly subsampled read pairs per strain.

Raw reads were trimmed for adapter sequences using Cutadapt v3.5 (7). Quality filtering was performed using fastp v1.0.1 (8), with the following parameters: --cut_right, --cut_right_window_size 4, --cut_right_mean_quality 18, --trim_poly_x, --poly_x_min_len 10, --n_base_limit 0, --low_complexity_filter, and --length_required 75. These options were adapted from the workflow described by Tourlousse et al. (9). A total of 1.5 million read pairs were randomly subsampled from each data set using SeqKit v2.8.2 (10), and the resulting reads were used for genome assembly. *De novo* genome assembly was conducted using SPAdes v4.2.0 (11), and genome annotation was performed using DFAST v1.3.9 (12). Genome completeness and contamination for the four assemblies were estimated using CheckM2 v1.0.2 (13). All assemblies showed 100% completeness, with contamination values ranging from 0.23% to 0.28%.

Draft genome assemblies ranged from 5.67 to 5.86 Mb with GC contents of 66.9%–67.0%. Average sequencing coverage values are summarized in Table 1. The genomes encoded 4,932–5,089 predicted protein-coding sequences, and full complements of tRNAs representing all 20 amino acids were identified in all genomes.

## Data Availability

The Whole-Genome Shotgun projects have been deposited in DDBJ/ENA/GenBank under accession numbers BAAKSV010000000, BAAKSW010000000, BAAKSX010000000, and BAAKSY010000000 for strains MAFF106604, MAFF211270, MAFF730132, and MAFF730133, respectively. The versions described in this paper are the first versions. The raw sequencing reads have been deposited in the DDBJ Sequence Read Archive under accession numbers DRR1065898, DRR1065899, DRR1065900, and DRR1065901, respectively. The data are associated with BioProject PRJDB41202.
